# Iconicity and transparency in Dutch Sign Language

**DOI:** 10.3758/s13428-026-02969-3

**Published:** 2026-03-13

**Authors:** Annika Schiefner, Floris Roelofsen, Beyza Sümer

**Affiliations:** 1 Institute for Logic, Language, and Computation, University of Amsterdam, Science Park 107, 1098 XG Amsterdam, The Netherlands; 2 Amsterdam Center for Language and Communication, University of Amsterdam, Spuistraat 134, 1012 VB Amsterdam, The Netherlands

**Keywords:** Dutch sign language, Iconicity, Transparency, Rating study

## Abstract

Iconicity in sign languages, visually motivated relationships between form and meaning, is closely tied to transparency, or the guessability of meaning based on its form. Yet, how to best measure this relationship remains contentious. This study compares iconicity ratings and transparency scores for 1412 lexical signs in Dutch Sign Language (Nederlandse Gebarentaal, NGT) across deaf NGT signers and Dutch and German non-signers. In this paper, we show that iconicity is perceived differently by the different groups of raters and replicate past findings that signers perceive signs from their own sign language as more iconic than non-signers do. No such differences are found between the two groups of non-signers. Our results show that Dutch non-signers’ performance on a transparency task is best predicted by iconicity ratings provided by participants from the same population and by non-signers more broadly. This has important implications for psycholinguistic research, as we show that there is no single “correct” population providing the best iconicity ratings. Instead, rating participants should be matched to the target populations on relevant dimensions to meaningfully predict outcomes in psycholinguistic studies. With this paper, we provide an extensive database of NGT signs with iconicity ratings and transparency scores, available as a reference database for Dutch Sign Language research. The database is aligned with glosses and item-IDs used in the NGT Signbank and Corpus projects, allowing for easy combination of our rating data with phonological and semantic information provided by those databases.

## Introduction

**Fig. 1:**
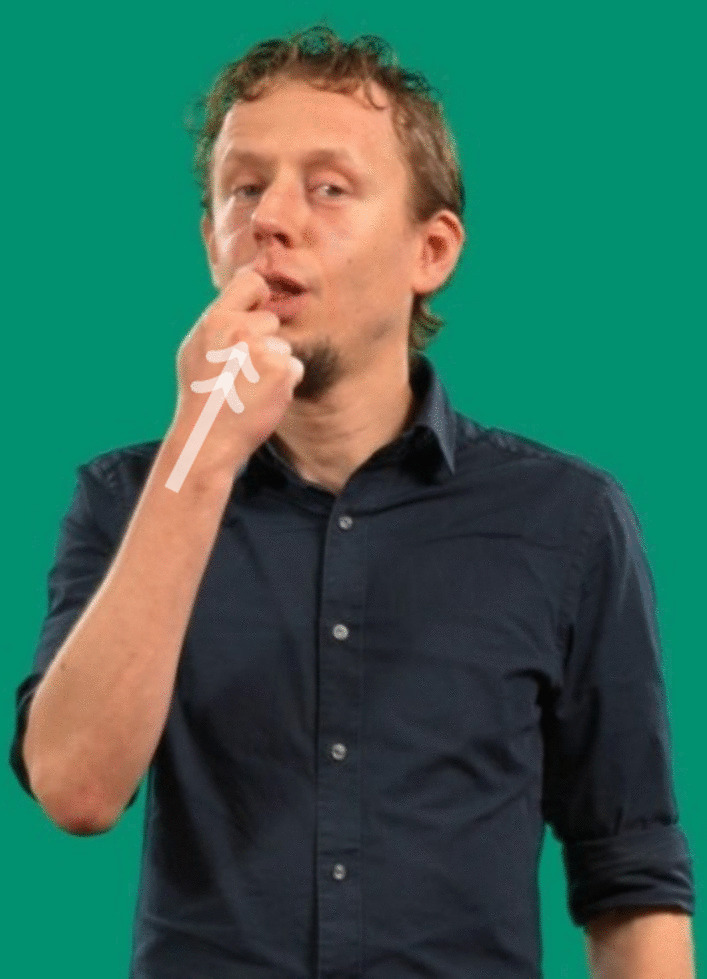
Still image of the NGT sign for eten(engl. *to-eat/food*). The *double arrow* indicates a repeated movement

In sign languages around the world, signs have often been found to look like what they mean (Bellugi, 1976; Klima and Bellugi, 1979). This property of signs to show some resemblance between the articulatory form and its meaning is called *iconicity* (Dingemanse et al., 2020; Perniss et al., 2010). This similarity is construed through a mapping process, in which the target meaning is schematized into relevant semantic features which are encoded through the articulators to create a linguistic form (Emmorey, 1651; Gentner, 1983; Taub, 2001). For example, signs for eat in most sign languages will depict moving food to the mouth in some capacity (see, for example, Fig. 1). Note that we use small caps to indicate sign language glosses. These are not intended to represent context-sensitive translations of the signs but provide an approximation of their lexical meaning.

Signs for eat may vary by how and with what utensils the food is handled, but the fundamental characteristics of the embodied experience of eating are shared across cultures and as such it is perhaps not surprising that their depiction would be similar. However, concepts differ in their propensity for such direct mappings (Masson-Carro et al., 2015; Ortega and Ozyürek, A., 2020; Schiefner, 2025), sign languages may differ in which mappings they select as a basis for lexical signs (Schiefner, 2025), and not all signs in sign languages are iconic to the same extent and in the same way (Caselli et al., 2017; Fuks, 2023; Klima and Bellugi, 1979; Ortega et al., 2025; Trettenbrein et al., 2021; Vinson et al., 2008). While spoken language research frequently takes iconicity to be a marginal phenomenon (but see also Blasi et al., 2016; Dingemanse et al., 2013; Fischer, 2014), sign language researchers have long acknowledged it as an important characteristic of the lexicon of sign languages (Capirci et al., 2022; Perniss et al., 2010; Taub, 2001). In fact, Boyes-Braem (1981) argues that about one in three lexical signs are iconic, suggesting, as have many others, that the visuo-spatial modality particularly strongly affords iconic mappings.

In this paper, we explore the perceived iconicity of approximately 1400 signs from Dutch Sign Language (*Nederlandse Gebarentaal*, NGT).^1^ We highlight differences and similarities between three groups of raters and show how these contribute to predicting the direct interpretation of iconic information in a transparency task with non-signers. This approach is based on taking two perspectives on iconic mappings. The description of *iconicity* compares a known form and known meaning in order to reconstruct the mapping process that relates them. The perspective of *transparency*, meanwhile, requires reconstructing possible target meanings from a known form, allowing for the exploration of the depictive potential of that form. We argue that the linguistic and cultural background of participants not only affects their perceptions and interpretations of iconicity (Occhino et al., 2017) but that these differences are likely to interact with how iconicity will affect outcomes in psycholinguistic studies that consider iconicity as a factor driving processing and learning.

Research into the specific effects of iconicity in language acquisition and processing yields mixed results (Gimeno-Mart’ınez, M., & Baus, C., 2022; Ortega, 2017). In this article, we argue that not only the domain and task in which effects of iconicity are investigated matter, but also the characteristics of the groups from which iconicity estimates are collected to predict these effects. Iconicity in signs has been found to create priming effects in deaf signers, facilitating sign detection when the primes highlighted the iconic link (Thompson et al., 2009; Vinson et al., 2008), yet other studies have not been able to replicate this effect (Bosworth and Emmorey, 2010; Emmorey et al., 2020; Ortega and Ostarek, 2021). Iconicity and overlap with iconic gestures have also been shown to facilitate sign production and recognition in hearing learners (Karadöller et al., 2024; Lieberth and Gamble, 1991; Mott et al., 2020; Ortega et al., 2019) and proficient hearing signers (Pretato et al., 2018), but it also appears to have hindering effects on phonological accuracy (Ortega and Morgan, 2015). Developmental studies have shown that iconic signs seem to be among the first to appear in the lexicon of deaf children (Karadöller, D.Z., Sümer, B., Ozyürek, A., 2025; Sümer et al., 2017; Thompson et al., 2012), though other studies have somewhat relativized these findings (Caselli and Pyers, 2017) and previous research has pointed out that the children themselves may lack the relevant world knowledge to interpret iconic form-meaning mappings in the first place (Meier et al., 2008). These studies highlight the effects of iconicity on sign language processing and learning.

However, these studies not only differ in the specific domains in which iconicity may show effects but, importantly, in who contributed the iconicity estimates that serve as a basis for investigation and how those estimates were collected. Studies that investigate the perception of iconicity as such have highlighted that participant demographics vary considerably in how they interpret iconic form-meaning links (Occhino et al., 2017). Occhino et al. (2017) show that signers find signs from their own sign language more iconic than those of another sign language they are not familiar with (for a replication in NGT, see also Omardeen, 2018). At the same time, even for unrelated and/or culturally distant sign languages, a notable proportion of concepts is represented through iconic signs that share the same symbolic basis, as observed across lexicostatistical investigations of sign language relatedness (Al-Fityani, 2006; Ebling et al., 2015; Guerra Currie et al., 2002; Kastner et al., 2014; Xu, 2006). This pattern also emerges in the study by Occhino et al. (2017), where the perception of signs from one’s own sign language also emerged for such items, suggesting that the specific encoding of iconicity and its interaction with phonological structure still increase the sense of familiarity and make a difference to deaf signers. Some studies also suggest that signers tend to rate signs as more iconic than non-signers (Griffith et al., 1981; Occhino et al., 2017; Trettenbrein et al., 2021), though results are not always consistent as Sehyr and Emmorey (2019) found that ASL signers rated signs as lower in iconicity than non-signers and Baus and Costa (2015) show very similar perceptions of the degree of iconicity between hearing non-signers and deaf signers in matching LSE signs with corresponding pictures. Overall, these findings suggest that signers are likely to overestimate the transparency of signs to non-signers, owing to their extensive experience with how iconicity is employed in the linguistic system of their language. As such, ratings provided by signers are unlikely to accurately predict effects in early learning by hearing non-signers, as they are overestimating the degree to which iconic mappings are available to these early learners.

One interesting question that emerges from this is whether similar differences would also be found between non-signers. Ortega and Ozyürek, A. (2020) show that Mexican and Dutch non-signers had similar biases for interpreting signs, which reflected similar underlying assumptions about iconic strategies, though Pizzuto (2000) found differences between Italian and other European non-signers, where Italians outperformed participants from other countries on a transparency task. Yet to our knowledge, no previous study has explicitly compared iconicity ratings of signs between groups of non-signers with different linguistic and cultural backgrounds. Indeed, Caselli et al. (2017) bring together ratings from non-signers who are fluent in English without reference to their individual cultural or linguistic background. In our study, we have therefore included a minimal cultural difference comparison, including both Dutch and German hearing non-signers. Their cultural and linguistic background is highly similar, speaking closely related languages and living in close proximity. As such, if we encounter systematic differences in their perception of iconicity, this would be a strong case for more carefully considering the linguistic and cultural background of raters when studying iconicity. If no such differences emerge, this would suggest that recruiting raters from at least somewhat diverging linguistic backgrounds is appropriate, as long as their familiarity with the sign language is kept constant. Future studies may then need to establish whether there are limits to this assumption and in what contexts differences may emerge.

Returning to the relationship between perceptions of iconicity in signers and non-signers, iconicity ratings provided by signers and non-signers are typically highly correlated, despite differences in the specific values assigned (Baus and Costa, 2015; Bosworth and Emmorey, 2010; Fuks, 2023; Sehyr and Emmorey, 2019; Thompson et al., 2009, 2010; Trettenbrein et al., 2021). Signers and non-signers thus appear to agree on the concepts that are higher or lower in iconicity, even though the specific distributions and mean ratings differ between groups. Across languages and studies, signers are more likely to show negative skew, with ratings clustered towards the higher end of the scale, while non-signers tend to show symmetrical or positively skewed rating distributions, with ratings clustered towards the lower end of the rating scale. However, it should be noted that there is substantial variation in the specific distributions found in different sign languages and studies, likely not only due to differences in rater characteristics and linguistic differences, but also related to details of the tasks and general rating behaviors of the different groups. For example, deaf Germans were less likely to use the higher end of the rating scale than deaf Americans in the study by Occhino et al. (2017), suggesting broader differences in scale use than could be explained by different perceptions of iconicity. This, then, suggests that while absolute estimates of iconicity are unlikely to generalize across populations, relative trends may reflect broader effects.

Considering the importance of linguistic knowledge when comparing signers from different sign languages and signers and non-signers, the question emerges whether parallel effects would also emerge when comparing non-signers with different linguistic and/or cultural backgrounds. Indeed, past research indicates that non-signers use their gestural repertoires to make sense of signs at first encounter and in the early phases of sign language learning (Ortega et al., 2019; Spruijt et al., 2023) and sign languages and gestures have been shown to share properties of iconic meaning making (Schiefner, 2025). It is therefore possible that non-signers, like signers, may find it easier to understand iconic mappings in a local sign language than in one from another cultural context. As past studies have elicited ratings only from non-signers within the same culture as the sign language or have not documented the specific cultural and linguistic background of raters, we do not know when and how differences in the linguistic background of non-signers would emerge. However, it stands to reason that such differences would be particularly likely to emerge only for sign languages from clearly distinct cultural contexts, and for culturally specific concepts and gestural emblems that are adopted into the sign language.

Iconicity can be measured in different ways, which can be categorized into studies that investigate how iconicity is realized in a given sign and studies that quantify the strength of the relationship between form and meaning (Dingemanse et al., 2020). The studies above all take the latter perspective, measuring the strength of iconicity. As we saw above, the linguistic and cultural background of the raters is highly relevant for this type of investigation. In the present study, we therefore investigate how different groups of raters perceive the iconicity of NGT signs. Such subjective ratings have a strong tradition in the investigation of iconicity in sign languages.

Collecting comparable ratings of the strength of iconic mappings is important for understanding the lexicon of different sign languages, with respect to the realization of form-meaning mappings. At the same time, it provides crucial information for conducting controlled psycho- and neurolinguistic studies with sign language materials. Subjective ratings have been shown to be a fruitful avenue for providing this type of information, as iconicity cannot be derived from corpus data.

A number of norming studies have been conducted to provide such datasets for British Sign Language (BSL) (Vinson et al., 2008), Spanish Sign Language (*Lengua de Signos Española*, LSE) (Gutierrez-Sigut et al., 2016), American Sign Language (ASL) (Caselli et al., 2017), Israeli Sign language (ISL) (Novogrodsky and Meir, 2020) and German Sign Language (*Deutsche Gebärdensprache*, DGS) (Trettenbrein et al., 2021). Other studies have collected iconicity ratings for stimulus selection and to study the effect of iconicity more directly (Baus and Costa, 2015; Bosworth and Emmorey, 2010; Fuks, 2023; Gimeno-Mart’ınez, M., & Baus, C., 2022; Griffith et al., 1981; Occhino et al., 2017; Ortega and Ozyürek, A., Peeters, D., 2020; Ortega et al., 2019; Sehyr and Emmorey, 2019; Thompson et al., 2009, 2010; Witz, 2024), covering a wide range of sign languages, adding NGT, Catalan Sign Language (*Llengua de Signes Catalana*, LSC), and Austrian Sign Language (*Österreichische Gebärdensprache*, ÖGS) to the previously mentioned. However, there are no previous attempts at creating a larger dataset of iconicity ratings for NGT. Smaller datasets with iconicity ratings by hearing non-signers have been collected in the context of other studies (Ormel, 2025; Ortega et al., 2019) and one study has collected iconicity ratings from deaf signers comparing their perception of iconicity in NGT and Chinese Sign Language (CLS) signs (Omardeen, 2018). However, the scope of these studies is smaller and they do not provide extensive interoperability with lexical resources for NGT, making cross-study comparisons difficult. The dataset for the present study directly addresses this gap, providing iconicity ratings for a large set of NGT signs collected from three distinct groups of raters.

As indicated above, iconicity is intricately related to the concept of *transparency*, the degree to which the relationship between a linguistic form and its meaning is evident to an observer. This is typically measured by asking non-signers to guess the meaning of signs, either by asking them to freely come up with translation suggestions or through single-choice paradigms. Griffith et al. (1981) already showed that hearing non-signers were largely unsuccessful at guessing the meaning of ASL signs in a free translation task. While they showed that transparency is a requirement for iconicity, it was not sufficient to account for iconicity’s effects on learning and linguistic processing. These findings have been replicated in a number of sign languages, e.g. Sehyr and Emmorey (2019) for ASL, Fuks (2023) for ISL, Trettenbrein et al. (2021) and Spruijt et al. (2023) for DGS. Pizzuto (2000) similarly show that iconic signs from Italian Sign Language are transparent to deaf and hearing people from six different countries to varying degrees, where familiarity with signing and Italian culture contribute to their success at interpreting iconicity. Guessing performance is significantly improved in forced-choice paradigms, however, where results often exceed chance accuracy (Breger, 1970), even in pre-school children from ages 3.5 years onwards (Tolar et al., 2008).

Occhino et al. (2017) shows that iconicity studies frequently conflate the two concepts by defining iconicity as an estimate of transparency in their instructions to raters. They argue that this conflation is problematic, as the assessment of iconicity strongly depends on the raters’ linguistic and non-linguistic knowledge and experience. However, it should be noted that a clean distinction between the two is likely to be unsuccessful. As Griffith et al. (1981) points out, the assumption of transparency is a requirement for iconicity ratings and intrinsic to the concept, as raters attempt to quantify the relationship between the form and its meaning. In the present paper, we consider iconicity ratings and transparency scores as two perspectives that capture different types of information about the same iconic mappings. While iconicity ratings provide information about the strength of the association between form and meaning, transparency scores and the variability of responses on the transparency task provide information on the depictive potential of the sign form, creating a possibility space that considers form against communicative, cultural, and motoric expectations.

As we have seen, iconicity and transparency are not mutually exchangeable concepts. For a full picture of the relationships between sign forms and their meanings, transparency scores provide additional information. On the one hand, the degree of semantic proximity between the translations provided by non-signers and the target translations offers a window into how evident mappings are to the naive recipients. Additionally, an analysis of the deviances from the target translations allows for a more nuanced understanding of how these non-signers attempt to decode the signs presented (for an example from DGS, see Trettenbrein et al., 2021).

**Fig. 2:**
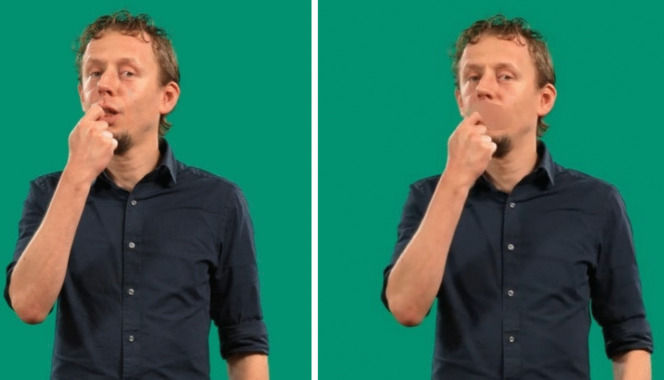
Still images of eten(engl. *to-eat/food*) with the mouth visible (left) and masked (right)

With this paper, we provide a database of approximately 1400 NGT signs with iconicity ratings from three groups of adult raters, transparency scores based on responses from Dutch non-signers, and information on response variability and certainty in the transparency task. Additionally, we provide experimental information to support future parallel dataset collection and facilitate large-scale data collection. To this end, we report detailed data on duration, dropout rates, and data quality evaluations. The signs in the database are associated with the Global Signbank NGT dataset (Crasborn et al., 2020; Klomp et al., 2024) through consistent ID-Glosses and unique numerical IDs, which allows for automatic extraction of phonological and other linguistic information. This large dataset can serve as a resource for future psycholinguistic studies in NGT.

## Methodology and materials

### Materials

A total of 1412 signs from NGT were included in this study. These signs represent everyday concepts, likely to be known by children in primary school, as they were recorded as part of a larger project developing a language learning game for primary school children. They cover a range of 25 semantic domains, as well as additional concepts classified as “other”. Signs were taken from the book “Mijn eerste 1500 gebaren” (engl. *My first 1500 signs* Gebarencentrum, 2012) and matched with the corresponding signs from the Global Signbank NGT dataset (Crasborn et al., 2020; Klomp et al., 2024).

Four deaf signers of NGT re-recorded the videos in studio conditions (two men, two women). The signers were professionals with ample experience in sign language video recordings. During the recording sessions, they provided input on the selected sign variants to ensure widely known variants were chosen. They were reimbursed for their efforts.

Recordings were produced from two angles, namely a frontal rendition while looking at the camera, and at a 45 degrees angle from the left. Every signer produced $50\%$ of the items frontally towards the camera and $50\%$ at the side angle. In the recording sessions, signers were presented with recordings of the target signs alongside the translation for the signs that would be presented in the game, through the SignCollect pipeline (Otterspeer et al., 2024). The presented recordings were directly imported from the NGT Signbank (Crasborn et al., 2020; Klomp et al., 2024) or recorded by the first signer based on the original signs of the Gebarencentrum (2012), if the variant was not available in the NGT Signbank, e.g., because it was a compound. Signers were asked to sign as they would when presenting the signs to children and to use mouthings and mouth gestures as they felt was appropriate to the sign.

For the iconicity and transparency studies, we used only items in which the signer faced the front camera and included signs produced by all four signers. In the game, children are presented with videos from both angles to provide better exposure to the three-dimensional nature of signs. The videos were processed for usage in the studies by replacing the background with a uniform single color background and blurring the mouth area of the video in the transparency task, to avoid any possibility of lip-reading (Pouw, 2025). We used two scripts for blurring the mouth, including and excluding hand detection, both of which are implemented in Pouw (2025). Subsequently, we manually checked which method provided the best compromise between keeping the manual information as visible as possible while maintaining occlusion of the mouth throughout the video. Mouthings and mouth gestures were visible in the iconicity task, as no part of the videos was blurred here (see Fig. 2 for an illustration of the difference between the unmasked and masked videos). All videos used in this study are available in the virtual appendix.

### Iconicity rating tasks

#### Participants

Participants were drawn from three distinct groups: deaf signers of NGT, hearing Dutch non-signers, and hearing German non-signers. Each group contributed ratings in an iconicity judgement task involving video stimuli.

The group “signers” consisted of 10 deaf adults who self-identified as NGT signers (5 women, 4 men, 1 ’other’). Participants reported using NGT “every day” ($n = 9$) or “most days” ($n = 1$). The age distribution was as follows: $18-29$ years ($n = 1$), $30-39$ ($n = 1$), $40-49$ ($n = 4$), $50-59$ ($n = 1$), $60+$ ($n = 3$). Five participants considered only NGT as their native language, one participant grew up orally with Dutch only, and four participants grew up bilingually with NGT and Dutch. Age of onset of learning NGT ranged from birth until 45 years of age, though only one participant reported learning NGT as an adult, and the median was at $= 2$ years. Except for the signer who learned NGT as an adult, all signers considered themselves to be native NGT signers. All participants also knew Dutch, regardless of whether they considered it a native language or not. Participants reported knowledge between 0 and 9 additional languages (median $= 3$). Most participants reported knowing international sign ($n = 8$) and/or English ($n = 8$). Since participants in this group were able to take breaks between blocks and to take part in the study across several day and participation is recorded between the first log-in and the end of the study, the time taken to complete individual lists cannot be reliably calculated. They provided ratings for 1400.9 items on average ($sd = 9.45$). These participants were recruited through the personal and professional networks of the research lab and affiliated team members. Due to the relatively small size of the signing community in the Netherlands, signers were invited to the lab for a full-day session, during which they rated the entire item set.

The group “Dutch non-signers” included 140 hearing adults (72 men, 65 women, one non-binary person and two with undisclosed gender), all of whom identified Dutch as their native language and reported no knowledge of NGT or another sign language. The age distribution was as follows: $18--29$ years ($n = 69$), $30--39$ ($n = 40$), $40--49$ ($n = 19$), $50--50$ ($n = 7$), $60+$ ($n = 5$). Six participants reported being bilingual in Dutch and English, while the rest had grown up monolingually. All participants reported knowledge of additional languages (median $= 2$, range: 1–6), with English known by all and German by 74 participants. Participants in this group needed an average of 15.67 minutes to complete the task and provided ratings for 105.99 items on average ($sd = 0.71$).

The group “German non-signers” also included 140 hearing adults (77 men, 60 women, one non-binary person, and two with undisclosed gender), all of whom identified German as their native language and had no knowledge of any sign language. The age distribution was as follows: $18--29$ years ($n = 54$), $30--39$ ($n = 40$), $40--49$ ($n = 27$), $50--59$ ($n = 6$), $60+$ ($n = 13$). Six participants reported growing up bilingually with German and another language. All but one participant reported knowing at least one additional language (median $= 1$, range: 0–7), always including English. No participants reported any knowledge of Dutch. Participants in this group took an average of 16.99 min to complete the task and provided ratings for 105.42 items on average ($sd = 3.06$).

Signers completed ratings for the entire set of items in-lab, while hearing non-signers from both countries were recruited online via the platform Prolific to complete ratings for subsets of items. These subsets contained approximately 100 unique test items and six control items for each participant. This implies the necessity of a larger pool of raters, more easily attainable for hearing than for deaf participants. Responses were screened for patterns of careless responses (e.g., uniform responses across trials) but no such patterns were identified. Online participants were compensated via the Prolific platform, while signers received an online voucher matched to the same hourly rate. A total of 10 participants claimed the task but then did not consent to participating in the study and 4 participants timed-out while in the survey. These candidates returned the task on the platform and were automatically replaced by other participants.

Each item was rated by an average of 9.95 participants per group ($sd = 0.22$, range: 7–10). Items with 5 or fewer ratings were excluded for that group. A small number of items served as quality control and were included in every list for hearing participants ($n = 2$) or in multiple lists ($n = 10$), resulting in a higher number of ratings for these items (see also Caselli et al., 2017). The latter were presented with their correct translations and, in half the cases, swapped, i.e., mismatched translations. These were excluded from the calculation of average ratings per item, but matched items are included in all further analyses.

#### Procedure

The 1412 total items were split into lists of approximately 100 signs. Participants were only allowed to complete one list, and all lists contained cross-matched catch items that were used to check response validity across lists. These catch-items consisted of two randomly selected signs (vader, *father*, and noot, *nut*) that occurred across all lists and a total of five pairs of randomly selected signs which were spread across lists so that for every list one of the pairs was presented with signs matched with their correct translations and one pair was presented with the translations swapped, i.e., mismatched between the two signs^2^. By comparing responses across lists, we could confirm consistency in ratings and verify that participants were not providing ratings as random, as ratings provided for the signs alongside their correct meaning would be expected to elicit higher ratings than when presented alongside a mismatched meaning.

Materials were presented in an online survey using Qualtrics. Participants first completed a section on informed consent, responded to a set of demographic questions, and then completed the ratings for a given list. Signs were presented individually and accompanied by a translation into Dutch or German. Participants were asked to rate how iconic they thought each sign was, i.e., how much it looked like what it means, on a scale from 1 (arbitrary) to 7 (iconic). Instructions were presented in NGT and written Dutch for deaf signers, and written Dutch or German with individual example videos from NGT for hearing non-signers, and were similar to instructions used in other rating studies (Novogrodsky and Meir, 2020; Perry et al., 2015).^3^ Participants were allowed to skip items, if they did not want to respond.

### Transparency task

#### Participants

Dutch hearing non-signers were recruited for the transparency study. The task was only offered to potential candidates who had not participated in any of the iconicity tasks, regardless of whether they had completed the task or not. This means that the sets of participants between the tasks are completely distinct. Responses were screened for patterns of careless responses (e.g., responding “no answer” to at least $50\%$ of items) and excluded and replaced by new participants if identified as such ($n = 1$). A total of 13 potential participants claimed the task but then did not give consent, and three participants were screened out. They were therefore excluded from the study and returned the task to be replaced by a different participant automatically. All participants were recruited through Prolific and received compensation for their time through that system. Participants in this group had to be native speakers of Dutch and not know any sign language.

A total of 140 hearing adults took part in the transparency task, distributed across 14 individual surveys (67 men, 72 women, and one who did not provide their gender), all of whom identified Dutch as their native language and reported no knowledge of NGT or another sign language. The age distribution was as follows: $18--29$ years ($n = 83$), $30--39$ ($n = 38$), $40--49$ ($n = 19$). Four participants reported being bilingual in Dutch and another language, while the rest had grown up monolingually. All participants reported knowledge of additional languages (median $= 2$, range: 1–5), with English known by all and German by 73 participants. Participants in this group needed an average of 31.2 min to complete the task and provided translations for 95.33 items on average ($sd = 10.7$).

Each individual item was translated by 9.13 participants on average ($sd = 1.08$). Items with 5 or fewer translations were excluded from analysis. A subset of items were used as quality control items for participants who provided translations for more than one list. These items, therefore, received substantially more translations, as they were included across multiple lists ($N = 12$). These were excluded from the calculation of average ratings per item.

#### Procedure

The total 1412 items were split into lists of approximately 100 signs. Participants were able to complete multiple lists, and all lists contained cross-matched catch items that were used to check response validity across lists.

Materials were presented in an online survey. The overall procedure was parallel to that of the rating study. In the task, signs were presented without translation, and participants were asked to type a translation into a text box. They were asked to type single words or short phrases and provided with an example, with simple instructions following the example of Ortega et al. (2019).^4^ If they could not come up with a translation, they were able to skip items without responding. The concept of transparency was not explicitly introduced to participants.

In addition to providing translations, participants were asked to rate their certainty for each response on a scale form 1 (not certain at all) to 7 (completely certain).

#### Transparency scores

In order to compute accuracy scores for responses in the transparency task, both responses and target translations were lemmatized and stop words were removed in R v. 4.4.1 (R Core Team, 2024)^5^ on Windows 11 x64 (build 26100) using the packages “spacyr” (v. 1.3.0, Benoit, 2023) and “stopwords” (v. 2.3, Benoit et al., 2021). Subsequently, we calculated semantic similarity based on semantic vectors for target translations and responses given (Yang, 2024). Scripts for this process were adapted and translated to R from Yang (2024) using unsupervised Dutch word embeddings (Schäfer, 2012; Tulkens et al., 2016). In these models, each word is defined as a vector in a multidimensional semantic space. Semantic distance is then defined as the angle between the response and the target vectors. Mean vectors were computed for multi-word items. The semantic similarity resulted in scores from 0 to 1, with scores of 1 indicating semantic identity. Semantic distances were aggregated into an average score for each sign, providing us with a measure of overall semantic similarity.

Note that this approach differs from other studies using manual coding, where correct translations and exact synonyms are considered correct, while any other response is considered incorrect (Ortega et al., 2019; Trettenbrein et al., 2021). Such binary coding systems can be turned into transparency scores by computing the proportion of correct responses. More directly graded scoring systems have shown to be very time-consuming to implement, though they have been suggested for naming tasks (e.g., Roomer et al., 2011). The approach taken in this paper, instead, would code only exact matches as correct, i.e., give them a score of 1, as even closely related synonyms are likely to show some semantic difference, resulting in scores of close to but not exactly 1. Contrarily, responses that are semantically related but not an exactly correct response would be scored as simply incorrect in traditional coding, while our approach allows us to quantify the semantic relationship, considering a response such as “house” to be a better response for shed than for the item box. This means that some translations that would be considered correct in manual scoring may be missed by our scoring when counting completely accurate responses, while the distribution of scores is likely to give more in-depth information about the relationships between targets and responses. We will discuss the implications of this approach in some more detail in the discussion.

#### Diversity of responses

To measure the diversity of responses provided for each sign, we calculated Shannon entropy scores of lemmatized responses by sign. Shannon entropy scores (*G*(*X*)) provide a measure of consistency and dispersion of responses based on the probability of each given translation observed for each sign *x* (*P*(*x*)) (see Eq. 1). Higher entropy values indicate greater diversity of responses, and lower values indicate greater consistency across participants.

1 $$\begin{aligned} G(X) = -\sum (P(x) * log(P(x))) \end{aligned}$$

In order to render the entropy scores comparable across signs, we normalized them against the number of unique responses given (Hausser, 2025). Normalizing entropy by item resulted in scores from 0 to 1, with scores of 1 indicating maximum entropy, i.e., every participant gave a different response, and 0 indicating that all participants gave the same responses. Entropy scores thus represent how diverse responses were for each sign.

### Analysis

Analyses were conducted using R v. 4.4.1 (R Core Team, 2024). To assess response quality, we compared iconicity ratings for control items between the matched and mismatched conditions to confirm that items received higher ratings when matched with the correct translation. Ratings for control items under the matched condition are included in the overall analysis of iconicity ratings by group.

We present descriptive statistics for the distribution of iconicity ratings and transparency scores for each rater group. We collapsed individual ratings into an average score per item by group and assessed the correlation between the iconicity ratings provided by different groups using Spearman’s rank correlation, taking into account that the rating scale only comprised 7 points and non-normal distributions of the rating data. We tested for between-group differences with Welch two-sample *t* tests. We also calculated the relationship between transparency scores and iconicity ratings provided by Dutch non-signers Kendall’s rank correlation, accounting for the non-normal distributions of the variables and the potentially non-linear nature of their relationship.

To assess whether participants were more confident about responses provided for signs that elicited more correct responses, we assessed the group-level association between average transparency scores and certainty ratings per item using Kendall’s rank correlation. At the level of individual responses, we computed whether individual certainty scores predicted the signs’ transparency, through a linear mixed-effects model.

Finally, to assess which factor most strongly predicted transparency of items, we fitted a linear mixed-effects model to predict transparency scores as a function of iconicity ratings provided by our three rater groups, mean certainty ratings, and normalized entropy scores with random intercepts for signs.

## Results

We asked participants from three groups, deaf NGT signers, Dutch hearing non-signers, and German hearing non-signers, to provide iconicity ratings for a total of 1412 signs. A distinct group of Dutch hearing non-signers provided translations for the same signs and rated how confident they were in their ratings.

**Fig. 3:**
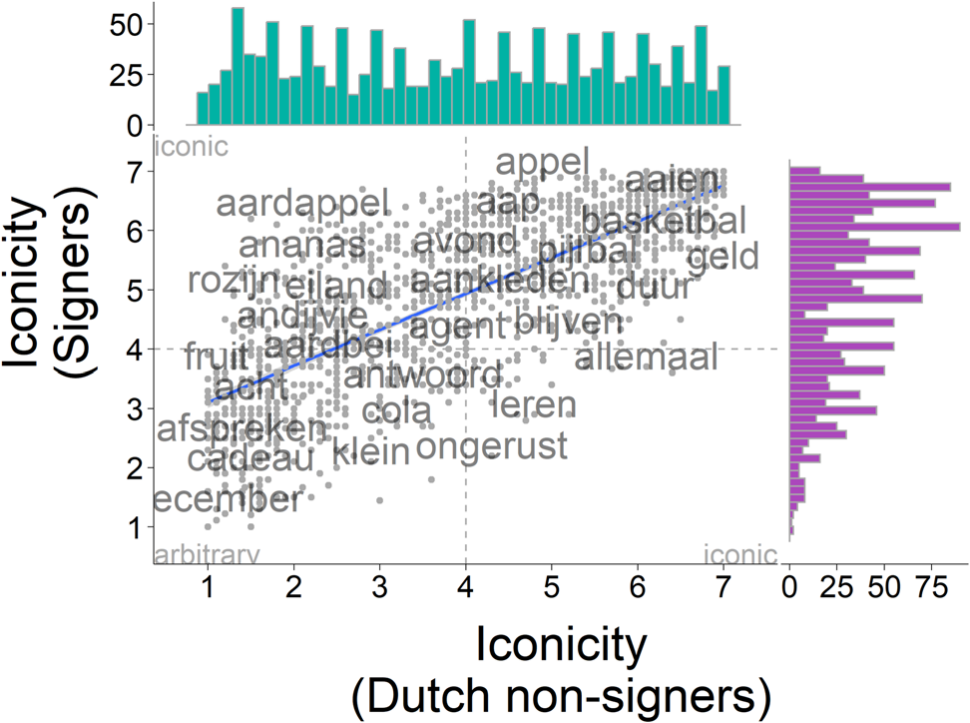
Correlation of iconicity ratings from deaf NGT signers (*purple*) and Dutch hearing non-signers (*turquoise*)

Quality checks revealed that participants rated signs with matching translations as significantly more iconic ($M = 3.52$, $SD = 2.02$) than signs with mismatched translations ($M = 1.71$, $SD = 1.08$, $t(1034.1) = -19.798$, $p <.001$). This indicates that the manipulation worked, as signs should be more iconic representations of what they really mean, and that participants were attentive to the relationships between the signs and translations presented.

### Relationships between iconicity ratings by different groups

**Fig. 4:**
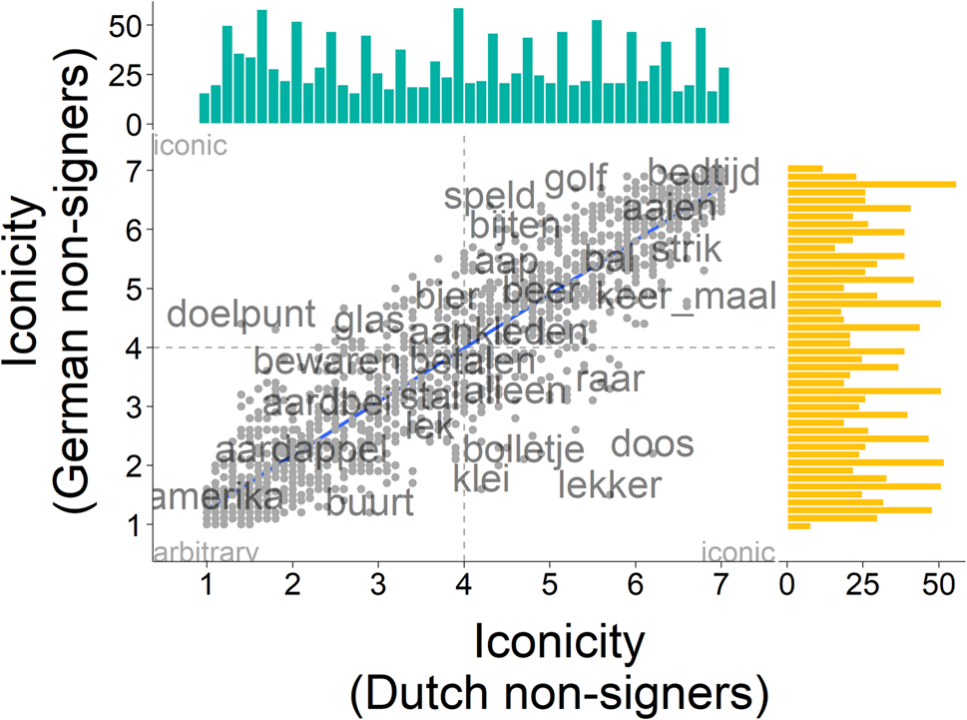
Correlation of iconicity ratings from Dutch (*turquoise*) and German (*yellow*) hearing non-signers

Three groups of participants contributed to the iconicity ratings. Deaf signers produced the higher iconicity ratings ($M = 4.90$, $SD = 1.43$) than Dutch non-signers ($M = 3.94$, $SD = 1.79$, $t(2694) = 15.705$, $p <.001$, Cohen’s $d = 0.59$, $95\%$ CI [0.52, 0.67]). There was no significant difference between Dutch and German non-signers ($M = 3.94$, $SD = 1.78$, $t(2805.9) = 0.07$, $p =.944$, Cohen’s $d = 0.003$, $95\%$ CI $[-0.07,0.08]$). This indicates that deaf signers of NGT perceived NGT signs as more iconic than did the non-signers (Fig. 3), while the difference in nationality and native language between Dutch and German did not show an effect (Fig. 4).

Comparing the different groups of raters on their individual ratings showed that mean ratings per concept were highly correlated across groups, as indicated by Spearman’s rank correlations. Non-signers were more highly correlated to each other ($\rho = 0.92$, $S <.001$, $p <.001$, see Fig. 4) than Dutch hearing non-signers were to the signers ($\rho = 0.77$, $S <.001$, $p <.001$, see Fig. 3). This indicates that while there were differences in the absolute average ratings across groups, the same items were perceived as high and low in iconicity, respectively, by raters from the three groups.

Ratings from both non-signer groups were evenly distributed across the entire rating scale, with no clear modes. Iconicity ratings by deaf signers, in contrast, were skewed towards the higher end of the rating scale, suggesting that signers considered more items as highly iconic than did non-signers. It is noticeable that while items that were considered high in iconicity by Dutch non-signers were also consistently considered as such by deaf signers of NGT, the reverse is not true (see Fig. 3).

### Relationship between iconicity ratings and transparency scores in Dutch non-signers

**Fig. 5:**
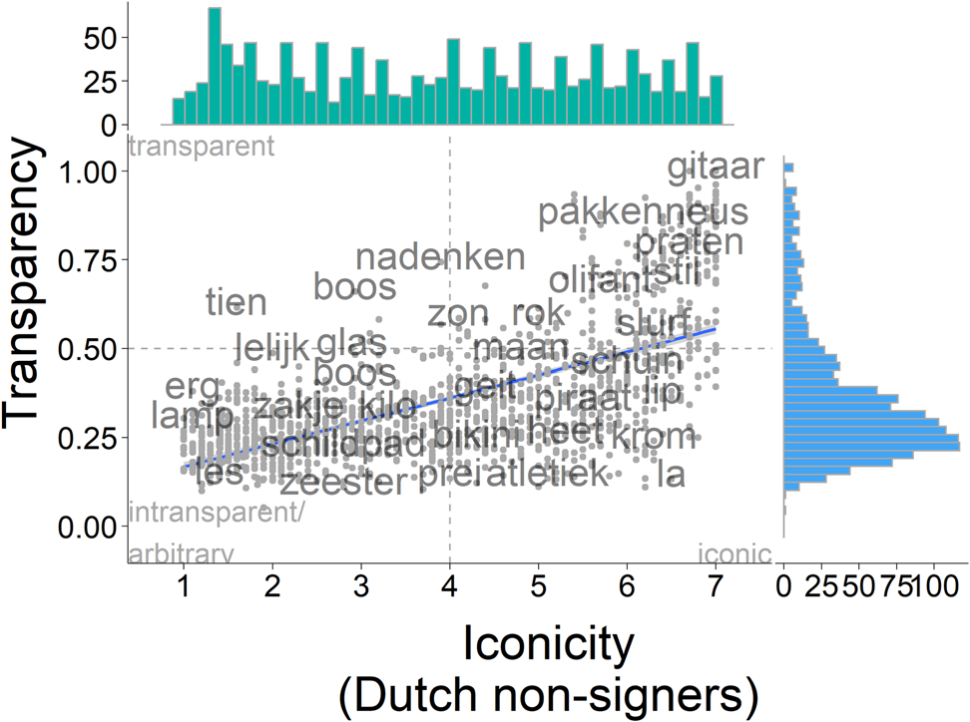
Correlation of iconicity ratings (*turquoise*) and transparency scores (*blue*) from Dutch non-signers

In line with previous research, participants were not consistently successful at guessing the meaning of the signs in our dataset. Across signs, transparency scores averaged at 0.34 ($SD = 0.19$) ranging from 0.05 to 1 (see Fig. 5). This indicates that signs differed in transparency.

**Fig. 6:**
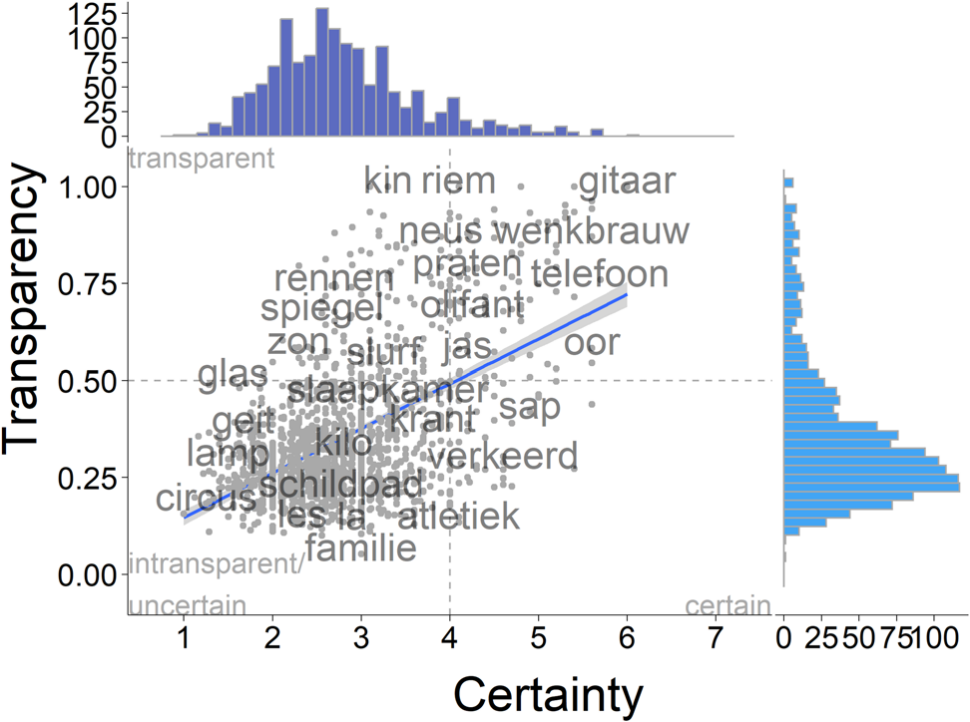
Correlation of certainty ratings (*dark blue*) and transparency scores (*blue*) from Dutch non-signers

Only three items consistently elicited correct responses (drie (*three*), vier (*four*), snijden (*to-cut*))^6^, while 973 items did not receive any correct responses and no semantic vectors were available for an additional 55 items, for which semantic distances could thus not be computed. In addition, some items did not elicit a full set of valid responses, yet all participants who gave responses gave an accurate response. These were gitaar (*guitar*, $n = 9$), riem (*belt*, $n = 8$), and kin (*chin*, $n = 7$). Out of the items for which more than half of the participants gave a valid response, 117 items received at least $50\%$ correct responses. Average certainty ratings were consistently low, even for those items that received consistently correct responses. gitaar (*guitar*) received the highest average certainty rating at 6, and 123 items received average certainty ratings of over 4, i.e. in the upper half of the rating scale (Fig. 6).

We found that transparency scores showed a medium-strength positive correlation with iconicity ratings provided by Dutch non-signers ($\tau = 0.45$, $z = 25.158$ $p <.001$), using Kendall’s $\tau$, which is sensitive to non-linear associations. This suggests that items that are rated as more iconic are indeed more transparent to non-signers from the same cultural context. Visual inspection suggests that the relationship between iconicity ratings and transparency scores may not be linear, with highly transparent signs receiving high iconicity ratings but not vice versa.

### Transparency and certainty

At a group level, certainty ratings were associated with transparency scores, such that participants provided higher average certainty ratings for items which yielded more consistently accurate responses ($\tau = 0.26$, $z = 14.355$, $p <.001$, see Fig. 6). To assess the effect at the individual response level, we fitted a linear mixed-effects model including random intercepts by sign and participant. The overall model fit was substantial (conditional $R^2 = 0.48$), but the fixed effect only accounted for $4\%$ of the variance (marginal $R^2 = 0.04$). Certainty significantly positively predicted transparency ($\beta = 0.03$, $t(11775) = 38.61$, $p <.001$, Std. $\beta = 0.19$), but is not a strong predictor on its own. Most of the variance in this model, instead, was accounted for by who responded to which item.

### Factors predicting transparency

To identify which of the collected measures best predicted the accuracy of responses on the transparency task, we fitted a linear mixed-effects model to predict mean transparency scores per sign as a function of iconicity ratings averaged across each of the groups of raters (signers, Dutch non-signers, German non-signers), mean certainty ratings, and normalized entropy, with random intercepts by sign. The model showed a strong overall fit (conditional $R^2 = 0.92$), providing substantial explanatory power. The fixed effects accounted for $54\%$ of the variance (marginal $R^2 = 0.54$).

Iconicity as rated by Dutch non-signers was significantly positively associated with transparency ($\beta = 0.04$, $p <.001$, $t(1378) = 7.77$, Std. $\beta = 0.39$), the strongest effects overall, indicating that transparency and iconicity ratings from the same demographic were most closely related. This was followed by a significant, strong negative effect of normalized entropy ($\beta = -0.44$, $p <.001$, $t(1378) = -13.45$, Std. $\beta = -0.29$), indicating that items with higher transparency received more homogenous responses from participants.

Iconicity ratings provided by German non-signers ($\beta = 0.02$, $p <.001$, $t(1378) = 3.46$, Std. $\beta = 0.17$) and mean certainty ratings ($\beta = 0.03$, $p <.001$, $t(1378) = 6.10$, Std. $\beta = 0.13$) were also significantly, positively associated with transparency but contributed less strongly to the model. Iconicity ratings provided by deaf NGT signers showed no reliable effect on transparency scores ($\beta < 0.001$, $p =.064$, $t(1378) = -1.85$, Std. $\beta = -0.064$). It should be noted that particularly iconicity ratings provided by Dutch and German non-signers were shown to be highly correlated, making their relative contributions difficult to accurately tease apart. These results suggest that intuitions about form–meaning resemblance among naïve non-signers are more closely aligned with transparency ratings than those of experienced signers, and that response variability (captured by entropy) is reliably associated with lower transparency scores, i.e., participants align more when giving responses that are semantically closely associated with the target meaning.

## Discussion

In this paper, we present norming data for iconicity and transparency for a total of 1412 signs from NGT. We collected iconicity ratings from deaf NGT signers, as well as hearing non-signers from the Netherlands and Germany. In addition, we computed transparency scores based on the translations provided by hearing Dutch non-signers for the same set of items.

We find that deaf NGT signers perceive the majority of signs as relatively iconic, resulting in a skewed distribution of iconicity ratings. Hearing non-signers from both countries provided ratings that were fairly evenly spread across the entire range, with no significant difference in rating scores between the two groups of non-signers. Ratings from the three groups were highly positively correlated, suggesting that the same items are rated as relatively more or less iconic by all groups, despite the differences in scale use. We also find that most items are not transparent to Dutch non-signers. Transparency scores computed as semantic similarity scores between the translations provided and the target translations for each item suggest that most items are extremely difficult to identify for naive observers. Only a handful of items consistently received translations that were good matches for the target translation. We find that transparency among Dutch non-signers is best predicted by iconicity ratings from raters within the same demographic. This suggests that iconicity ratings are most relevant to predicting sign language processing and learning effects when raters are matched to the target demographic on relevant characteristics.

### Distribution of iconicity ratings

We find that iconicity ratings provided by signers of NGT are skewed towards the iconic end of the rating scale, suggesting that these signers perceive the majority of signs from their sign language as relatively iconic. This is in line with previous studies which have reported similar patterns for a variety of languages (e.g., Trettenbrein et al., 2021, for DGS, Ortega et al., 2025 , for DGS and BSL). Meanwhile, ratings from hearing non-signers in our study showed a remarkably even distribution of iconicity ratings, ranging from arbitrary to fully iconic, with no clear bias towards either end of the scale. Ratings between Dutch and German non-signers were also highly positively correlated and showed very similar distributions. While past studies have shown the same effect of signers rating signs as more iconic than non-signers across a variety of languages, these studies have typically reported a skew towards the lower end of the iconicity scale for hearing non-signers (Caselli et al., 2017; Griffith et al., 1981; Klima and Bellugi, 1979; Ortega et al., 2025; Trettenbrein et al., 2021). Only one study has reported the opposite effect, eliciting higher iconicity ratings from non-signers than from signers for ASL (Sehyr and Emmorey, 2019).

It is possible that this is a methodological effect, stemming from the selection of concepts that children are likely to know, as some studies have suggested that at least the very early vocabulary of children may be more iconic than the overall lexicon of sign languages (Novogrodsky and Meir, 2020; Thompson et al., 2012) and that signs acquired early received the highest iconicity ratings (Vinson et al., 2008). However, even if such a methodological bias exists in our dataset, the relative difference between signers and non-signers persists.

These persistent differences between signers and non-signers in how iconic they find individual signs, while agreeing on which signs are more or less iconic overall implies that the linguistic knowledge of signers on how iconicity is systematically used in their sign language affects the embedding of interpreting iconicity as part of the system. Signers are able to understand and reconstruct systematic use of iconicity, e.g., involvement of classifier handshapes in lexical signs, in ways that are not accessible to non-signers (Occhino et al., 2017). The specific context in which iconicity is interpreted by individuals is thus highly relevant to how iconicity would be expected to affect learning and processing in different groups. There is also ample inter-individual variation in the perception of iconicity in both signers and non-signers (Occhino et al., 2017), yet the way that iconicity can be construed for each sign seems to provide guide rails for these interpretations and drive group-level effects that differentiate groups of raters. Both signers and non-signers appear to tap into this same latent potential for iconicity (Perlman and Woodin, 2021).

In this context, it is interesting that we observe such minimal differences between Dutch and German hearing non-signers. While one might expect that cultural variation or subtle differences in the semantic features of near-translation equivalents and associated gestural repertoires could influence perceptions of iconicity (Occhino et al., 2017; Ortega and Ozyürek, A., Peeters, D., 2020), our data suggest that such differences may be minimal and do not exceed inter-individual variation within language communities. Specifically, members of language communities that are culturally and linguistically similar to the target population appear to perceive the iconicity of unknown signs in highly similar ways. This suggests that perceptions and construals of iconicity are not tightly bound to language-specific preconceptions, but rather reflect broader world knowledge and cognitive biases shared at least by speakers of related languages from neighboring cultures.

This finding has practical relevance, particularly for studies requiring the creation of large datasets, as presented in this paper. Recruiting large numbers of participants who meet narrow criteria, e.g., Dutch speakers active on Prolific and willing to engage in a $20--30$-min task, proved difficult for the total of 280 Dutch participants necessary for the present study. If raters from a wider geographical area but similar cultural-linguistic backgrounds can provide reliable ratings, this facilitates data collection immensely. In fact, Caselli et al. (2017) implicitly follow this logic as they placed no geographic restrictions on their non-signer participants, requiring only that they be English speakers. While other studies have typically recruited participants with a specific linguistic background, this appears to be more a matter of convenience than a theoretically grounded decision. More research should establish the limits of such approaches, particularly considering how suitable ratings from different groups are for predicting outcome variables, such as effects on learning or language processing in different target groups (Gimeno-Mart’ınez, M., & Baus, C., 2022). The dataset in this study provides the necessary resource for such psycholinguistic studies and is suitable for cross-linguistic comparisons with other sign languages, given the phonological information available from the Global Signbank NGT dataset (Crasborn et al., 2020; Klomp et al., 2024).

### Semantic information in iconic mappings and transparency scores

Iconicity ratings of Dutch non-signers and transparency scores computed from the translation attempts of distinct participants from the same demographic showed a medium-sized, positive correlation. While iconicity ratings were evenly spread in this group, most items turned out to score low on transparency, in line with past research that suggests that non-signers perform very poorly on free text transparency tasks (Fuks, 2023; Klima and Bellugi, 1979; Lieberth and Gamble, 1991; Ortega et al., 2019; Sehyr, 2022; Trettenbrein et al., 2021). As has been shown for other languages and in smaller samples of NGT lexical items, the small subset of items that are highly transparent also receives high iconicity ratings, yet iconicity ratings are not a sufficient predictor for transparency. Most signs, even those with high iconicity ratings, are difficult to identify for hearing non-signers.

Operationalizing transparency, as we have done, by investigating the distance between semantic vectors for the target word and the provided translation, represents semantic associations based on spoken language characteristics and occurrence. However, when inspecting responses provided by participants, it quickly becomes clear that most answers are not semantically unrelated to the semantic features of the target referent depicted in the iconic mapping. Instead, participants appear to be correctly identifying relevant form-meaning associations below the lexical level but under- or overgeneralize in their interpretation of the lexical meaning. For example, the sign timmeren (*to do carpentry, to build, to hammer*) imitates the handling of a hammer. Some responses interpreted the movement of the hand as swinging a bell in the responses “bel ”(*bell*) and “klepel” (*bell clapper*). While it is clear that these responses are misinterpreting the sign with respect to the target meaning, it is not true to say that non-signers are unable to identify relevant characteristics of the form and interpret them in the context of a possible iconic mapping. Even more strikingly, responses such as “hameren” (*to hammer*) or “(aan-)kloppen” (*to knock*) result in relatively low semantic similarity ratings based on Dutch written corpus data, yet the embodied similarity between the associated actions is immediately obvious and touches on the core of the iconic mapping. Semantic vectors derived from multimodal corpus data, as suggested by Hagoort (2024), may be better suited to reflect such patterns, but they are not yet available.

This highlights a phenomenon of iconic mappings described by Mittelberg (2014) for gestural representations. She argues that “[g]estures, like most other signs, tend to be partial representations and thus metonymic” (2014, 1747). This means that in interpreting unknown signs, non-signers still need to fill in the mapping process from the partial representation to a full, lexical item. In this process, they may diverge from the mapping process that underlies the true target translation. The specific translations provided by our participants are in line with their suggestion that observers will more readily interpret signs as relying on external metonymy, pantomiming in coordination with virtual objects, than internal metonymy, where body parts stand in for objects (Mittelberg, 2014 1761). It is thus not entirely fair to the participants to argue that they are unable to reconstruct the iconic mappings in the signs. A better description might be that they are correctly identifying the potential iconic mappings and extrapolating possible lexical meanings from those. However, given the multitude of possible iconic mappings afforded by a single manual representation, their translation attempts may not select the “correct” interpretation. Under this interpretation, analyzing the variability of responses gives us insights into the density of potential iconic mappings for a given form, potentially identifying items for which sign languages will have a higher need for disambiguation or may associate a wider range of lexical meanings with a single manual form.

### Transparent items

Consistent with past research, only very few items elicited correct responses from all participants, while almost $70\%$ of signs were never guessed correctly. The items that were consistently correctly identified were number signs under five (signs over 5 are more difficult to guess, due to the one-handed counting system of NGT), and the verb to cut. For another set of items, not all participants responded, but those who did correctly identified the signs for guitar, belt, and chin. All of these items also received very high iconicity ratings with average ratings in all groups $>6$. There is no clear similarity between these items, as they represent the concepts in question through different types of iconic mappings, and especially the signs for belt and chin would be unlikely to be considered gestural emblems.

Interestingly, besides guitar, violin and flute also received mean transparency scores above 0.9, with nine and seven correct responses, respectively. It seems like these are highly prototypical instruments associated with distinctive movement patterns. It is likely that this distinctiveness of associated movements and shape information contributes to transparency more generally, though it is a property difficult to capture in a categorical coding system (but see Thompson et al., 2020). As objects and actions share similar motoric representations, they become less distinguishable in gestural and signed representations. While sign languages can encode subtle differences by exploiting different iconic strategies (Schiefner, 2025), non-signers have no linguistic system knowledge for interpreting these representations other than their gestural repertoires (Ortega et al., 2019). The effects of phonological neighborhood densities in signed or spoken languages may provide a model for possible effects on the identifiability of such signs in learners and non-signers (Thompson et al., 2020).

On a methodological note, these items also highlight in what ways the present approach is stricter than traditional manual coding approaches and where it allows for a more nuanced perspective. Where a manual coding approach may consider responses such as “viool spelen” (engl. *playing the violin*) as correct responses, an approach using vector comparisons computes a somewhat lower score and does not count such deviances as correct responses. At the same time, similar questions also come up in manual coding. For example, Ortega et al. (2019) define answers that include a change in part of speech or verbs that contain the correct argument as correct answers, but not verbs that are applicable but not exclusive to the target word as incorrect. From the responses given in the survey on the instruments mentioned above, this means that responses like the above mentioned *playing the violin* would presumably be coded as a correct response, while “instrument spelen” (engl. *playing an instrument*) would be rejected as a correct response for flute. As such, taking a perfect match as the cut-off for correct responses is likely to result in a stricter coding than would traditionally be employed, though the graded nature of the transparency scores seem to capture important intuitions about the semantic relatedness of responses and targets that are also targeted by manual coding. It is also important to note that the word embeddings are based on the Dutch words. This is likely to be a good representation of the semantic understanding of Dutch non-signers, but may not accurately reflect the semantics of the signs. However, semantic embeddings for signs are not currently available for any sign language, and the question of whether these would include semantic dimensions that do not emerge in spoken languages thus remains open for the time being.

The number signs for three and four are likely to serve as emblems in spoken Dutch, though the frequent misinterpretation of eight and nine as depicting the same numbers suggests that the specific fingers extended for showing numbers are not relevant in Dutch co-speech gestures. This supports the notion that gestures are more holistic and less clearly defined in their articulation, showing what Kita et al. (2025, 2014) call “sloppy articulation”.

### Predicting transparency

Transparency scores capture how easily non-signers can infer a sign’s meaning from its articulatory form. They are thereby most relevant to initial learning, where immediate recognition can bypass explicit instruction and allow learners to rely on it in future encounters. Iconicity ratings, meanwhile, are more relevant to ongoing learning processes, in which learners need to consolidate links between form and meaning for reliable retrieval. Of these tasks, transparency scores are more effortful to collect, as participants take longer translating items than providing simple ratings and the task produces more noisy data, which is difficult to assess and turn into meaningful quantitative information, in particular, when taking traditional approaches of manually coding the accuracy of open-cloze answers employed in many studies (see, for example, Ortega et al., 2019; Trettenbrein et al., 2021). This makes iconicity ratings a useful shortcut for estimating difficulties that learners may encounter in understanding and remembering signs, but poses the question of how accurately they can predict transparency.

We found that transparency was best predicted by iconicity ratings provided by the same demographic of Dutch hearing non-signers. This indicates that the way non-signers perceive and interpret iconic mappings is closely related to how they process unfamiliar signs without translations. At first exposure, learners acquiring sign language items must derive meaning from visual input and potentially the linguistic content, if embedded in a more natural learning environment. Signers, unlike non-signers, are able to exploit their knowledge of how sign languages systematically incorporate iconicity in their linguistic structures as they provide iconicity ratings (Occhino et al., 2017). Iconicity ratings are therefore most informative in predicting behaviors on learning and translation tasks, when raters and task participants share similar linguistic resources. In our specific example, non-signers only compare the articulatory form they are presented with to the meaning suggested, while signers also consider alternative possible translations of the sign, alternative signed translations of the Dutch word, and wider conventions on how iconic structures are used in NGT morphology and to structure semantic networks. They commented on whether a sign was a “good” iconic sign, by comparing it to other signs that they thought were more or less iconic and the way the iconic mapping worked. The non-signers in the transparency task have even less information, relying only on articulatory form in their attempt to reconstruct meaning.

Comparisons across groups of raters underscored this influence of linguistic knowledge. Transparency ratings provided by Dutch and German non-signers correlated strongly and showed no systematic differences, reflecting similarities in the cultural and linguistic repertoires available with respect to interpreting the unknown sign language. By contrast, deaf signers diverged from both groups, providing consistently higher ratings. This is in line with past research, which found similar effects when comparing ratings between signers and non-signers in different sign languages (Griffith et al., 1981; Occhino et al., 2017; Trettenbrein et al., 2021). Notable exceptions from the high agreement between Dutch and German raters were lekker (*tasty*) and doos (*box*). In the case of lekker, there is a gestural emblem in spoken Dutch with the same meaning, creating a high familiarity for non-signers interpreting this sign. A similar emblem is not available in German. This underlines the importance of linguistic and communicative repertoires in the interpretation of iconicity, where familiarity clearly creates a feeling of recognition that is interpreted as iconicity by observers. In the case of doos, the semantic extensions of the Dutch and German cognates do not entirely overlap and the translation given to German raters, though not unrelated, was not the optimal translation of the type of object described by the sign. Together, these findings highlight how linguistic and world knowledge create the cognitive backdrop against which effects of iconicity emerge. Matching iconicity rating participants to the populations studied in learning and processing experiments is therefore crucial for creating valid predictions on how iconicity may affect psycholinguistic outcomes.

Items with higher transparency elicited more homogenous responses and higher certainty ratings, indicating that participants not only converged on the same interpretations but also had a sense of when their guesses were correct. This convergence is not trivial, as responses that were close matches but not entirely correct could, in principle, be highly diverse, or signs could have been highly iconic representations of a meaning other than the lexical meaning given, leading to systematic misinterpretations. Instead, errors were highly variable and associated with low certainty, suggesting that signs tend to be lexicalized for their most iconic interpretation. Participants thus displayed metacognitive sensitivity to their own translation success, even though individuals differed in how they used the certainty rating scale. Together, these results show that transparent items are characterized by low entropy and high certainty. These findings are reminiscent of observations by Yang (2024), who shows similar effects in the interpretation of iconic gestures. In their paper, Yang (2024) argue that these patterns reflect a pressure for communicative efficiency, which might also contribute to the role of iconicity in a sign language lexicon. Our findings support arguments about the structuring role of iconicity in the lexicon of sign languages and its importance for understanding how form-meaning mappings are perceived and interpreted by non-signers.

### Usage of the database

The present paper not only provides an analysis of iconicity ratings for a large number of NGT signs but also a database of studio-quality recordings of these signs with possible translations to written Dutch. The stimulus videos are available for reuse for academic purposes under a CC BY-NC 4.0 license, while the analysis materials are licensed under CC BY 4.0 (for details and access see “Availability of data, materials and code”). This multimodal database, combining video data, links to corpus-based information and phonological annotations via the NGT Signbank (Crasborn et al., 2020; Klomp et al., 2024), and psychometric information on the iconicity and transparency of these items, is intended to serve as a reference for future psycholinguistic studies of NGT. The signs presented are selected to be relatively frequent and well known to children and adults alike. As such, they provide a good starting point for sign learning studies in children but also adult learners of NGT. This database can thus serve to facilitate controlled psycholinguistic experiments and learning studies, by compiling comparable and reliable estimates of iconicity and transparency for a large set of signs. Parallel endeavors mapping the relationship between form and meaning at scale for different sign languages are currently underway, illustrating that such efforts are timely and sought after across the field (see, for example Trettenbrein et al., 2025; Morgan, 2023). Existing studies in other sign languages illustrate the potential of such databases (see, for example, Vinson et al., 2008; Trettenbrein et al., 2021; Caselli et al., 2017), providing the foundational data to measure the effects of iconicity, transparency, and phonological relationships on sign language processing, production, and acquisition.

## Conclusion

In this paper, we have provided evidence that the linguistic and cultural background of participants not only affects their perceptions and interpretations of iconicity (Occhino et al., 2017) but that these differences are likely to interact with how iconicity affects outcomes in psycholinguistic studies on sign language processing and learning. We show that transparency scores are best predicted by iconicity ratings provided by raters from the same demographic, i.e., hearing non-signers with the same language background, though the specific language background of the non-signers played a negligible role. The lack of experience with NGT signs and missing linguistic framework for interpreting these signs were most important to outcomes on the transparency task.

Overall, our findings suggest that there is not one “correct” population which provides the purest or best iconicity ratings. Instead, rating populations should be matched to the target populations to meaningfully predict outcomes in psycholinguistic studies. This permits for specific predictions of how iconicity ratings would contribute to outcomes on psycholinguistic processing and learning tasks, as they reflect mechanisms of how iconicity is perceived.

## Data Availability

Data, analysis scripts are available through our open access repository on OSF, licensed under CC BY 4.0. Identifier: https://doi.org/10.17605/OSF.IO/U7RKF Videos of the signed concepts in NGT alongside signed and written instruction materials are available through a collection on Figshare, licensed under a CC BY-NC 4.0. DOI: 10.21942/uva.c.8301958

## Footnotes

1 Past research has used the term Sign Language of the Netherlands as an English translation of the name of Nederlandse Gebarentaal. However, following preferences from the Deaf community, this paper uses the term Dutch Sign Language and the abbreviation NGT, borrowed from written Dutch (Gilchrist, 2024).

2 Pair 1: boos (*angry*), verf (*paint*); Pair 2: poffertjes (*small pancakes*), gedicht (*poem*); Pair 3: haten (*to-hate*), overdrijven (*to-exaggerate*); Pair 4: sticker (*sticker*), vloer (*floor*); Pair 5: brand (*fire*), spruitjes (*brussels sprouts*)

3 The instructions are available alongside an English translation in the supplementary materials.

4 The instructions are available in the materials.

5 A full list of packages is available in the analysis script.

6 It is worth mentioning here that NGT uses a one-handed counting system, where the numbers from one to four count from the index finger, while the numbers from six to nine start counting from the thumb. This is not the case for Dutch gestures, so, number eight, for example, was usually misinterpreted as number three.
